# Let-7b, miR-29b, and miR-125b as Potential Biomarkers for Differentiating Canine Mammary Carcinoma Histological Types

**DOI:** 10.3390/ani15010020

**Published:** 2024-12-25

**Authors:** Tiago Ferreira, Francisca Dias, Ângela Alves, Adelina Gama, João F. Mano, Paula A. Oliveira, Rui Medeiros

**Affiliations:** 1 Centre for the Research and Technology of Agro-Environmental and Biological Sciences (CITAB), University of Trás-os-Montes and Alto Douro (UTAD), 5000-801 Vila Real, Portugal; pamo@utad.pt; 2 Institute for Innovation, Capacity Building and Sustainability of Agri-Food Production (Inov4Agro), University of Trás-os-Montes and Alto Douro (UTAD), 5000-801 Vila Real, Portugal; 3 Molecular Oncology and Viral Pathology Group, Research Center of IPO Porto (CI-IPOP)/RISE@CI-IPOP (Health Research Network), Portuguese Oncology Institute of Porto (IPO Porto)/Porto Comprehensive Cancer Center (Porto.CCC), 4200-072 Porto, Portugal; francisca.carvalho.dias@ipoporto.min-saude.pt (F.D.); angela.m.alves@outlook.pt (Â.A.); 4 Department of Chemistry, CICECO—Aveiro Institute of Materials, University of Aveiro, Campus Universitário de Santiago, 3810-193 Aveiro, Portugal; jmano@ua.pt; 5 School of Medicine and Biomedical Sciences (ICBAS), University of Porto, 4050-513 Porto, Portugal; 6 Animal and Veterinary Research Centre (CECAV), University of Trás-os-Montes and Alto Douro (UTAD), 5000-801 Vila Real, Portugal; agama@utad.pt; 7 Associate Laboratory for Animal and Veterinary Sciences (AL4AnimalS), University of Trás-os-Montes and Alto Douro (UTAD), 5000-801 Vila Real, Portugal; 8 Faculty of Medicine, University of Porto (FMUP), 4200-319 Porto, Portugal; 9 Research Department of the Portuguese League against Cancer—Regional Nucleus of the North (Liga Portuguesa Contra o Cancro—Núcleo Regional do Norte), 4200-177 Porto, Portugal; 10 Virology Service, Portuguese Institute of Oncology (IPO), 4200-072 Porto, Portugal; 11 Biomedical Research Center (CEBIMED), Faculty of Health Sciences of the Fernando Pessoa University, 4249-004 Porto, Portugal

**Keywords:** canine, CMTs, diagnosis, dog, microRNA, RT-qPCR

## Abstract

MicroRNAs (miRNAs) are short non-coding RNA molecules involved in post-transcriptional control. They are closely related to cancer-associated processes, making them promising candidates for diagnostic and prognostic biomarkers, as well as targets for cancer therapy. This study investigated the expression levels of let-7b, miR-29b, miR-125b, and miR-146 in normal and neoplastic canine mammary gland samples by quantitative reverse transcription polymerase chain reaction (RT-qPCR). Furthermore, the miRNA profile of the tumors was compared to adjacent mammary glands and to normal control mammary tissues. MiR-29b and miR-125b were downregulated in both benign and malignant lesions compared to the control, while let-7b was significantly downregulated only in malignant lesions. Additionally, miR-29b was upregulated in the malignant group compared to the benign group. Regarding histological type analysis, miR-125b was downregulated in simple carcinomas compared to complex carcinomas, whereas let-7b and miR-29b were upregulated in complex and simple carcinomas compared to the other subtypes, respectively. MiR-29b has been shown to be able to differentiate malignant from benign, while miR-125b can discriminate simple carcinomas from complex carcinomas. In addition, let-7b, miR-29b, and miR-125b, together, can discriminate simple and complex histological types from other histological types. In conclusion, the present study demonstrated that the expression of these miRNA changes during the tumorigeneses process, representing an epigenetic mechanism to sustain canine mammary tumor development, and identified these miRNAs as promising diagnostic biomarkers.

## Abstract (plain language summary)

A panel of four microRNAs (miRNAs) was investigated in normal and neoplastic canine mammary gland samples using quantitative reverse transcription polymerase chain reaction (RT-qPCR). The relationship between these miRNAs and histopathological features, including benign and malignant lesions, as well as different histopathological types (simple carcinoma, complex carcinoma, and other subtypes), was explored. MiR-29b was identified as a potential biomarker of malignancy, as its levels were elevated in malignant samples compared to benign samples. Furthermore, let-7b, miR-29b, and miR-125b, together, appear to have the potential to discriminate among the three histological types.

## 1. Introduction

Epigenetics is the study of changes in organisms that result from modifications in gene expression rather than alterations in the genetic code itself. These changes affect chromatic dynamics and, consequently, gene regulation and expression [1,2,3]. The main mechanisms of epigenetics can be divided into four types: DNA methylation, histone variants, histone post-translational modifications, and non-coding RNAs (ncRNAs) [4]. This last type, in particular, encompasses a class of RNA sequences that are transcribed but do not encode proteins [5]. NcRNAs are often grouped according to their size: small ncRNAs (<200 nucleotides) and long ncRNAs (>200 nucleotides) [6]. MicroRNAs (miRNAs) are small ncRNA molecules with an average length of 22 nucleotides and are involved in post-transcriptional gene regulation in diverse biological contexts [7]. In cancer, they can act as oncogenes or tumor suppressor genes [8]. The deregulation of miRNA expression in both tumor development and progression has made these molecules an important biomarker in cancer research, namely in breast cancer, where they were described as potential diagnostic and/or prognostic biomarkers [9,10,11]. In veterinary medicine, miRNA research is still an emerging field. However, it has been receiving an increasing level of attention, as ongoing research suggests that microRNAs can serve as valuable tools for diagnostic and prognostic biomarkers in this field as well [12]. Canine mammary tumors (CMTs) are the most prevalent neoplasms in intact female dogs, and approximately 50% of these tumors are malignant [13]. CMTs constitute a heterogeneous group of tumors characterized by variations in morphology and biological behavior. CMTs exhibit several similarities to human breast cancer (HBC), establishing them as a valuable translational model for breast cancer research [13]. One of these characteristics is their genetic similarity, namely miRNA deregulation, which can be very useful when studying potential biomarkers [14]. In CMTs, research into miRNA expression has been relatively limited; nevertheless, existing studies suggest that miRNA expression patterns in CMTs resemble those of HBC [15,16,17]. In the present study, we analyzed a panel of four miRNAs (let-7b, miR-29b, miR-125b, and miR-146) in canine mammary tumors (CMTs) and in normal/adjacent mammary gland tissues that were previously associated with diagnostic and/or prognostic importance in HBC [18,19,20,21]. Our goal was to evaluate the changes in expression levels of these miRNAs in CMT samples and assess their potential to discriminate between the defined groups and their consequent applicability in the veterinary field.

## 2. Materials and Methods

### 2.1. Ethical Statement

This study was conducted in accordance with the European Directive (2010/63/EU). All procedures were performed after ethical approval by DGAV (Direção Geral de Agricultura e Veterinária, Lisbon, Portugal) with reference number 004582.

### 2.2. Animal Population, Sample Collection and Histopathology

Forty-nine mammary tumor samples from 31 female dogs were surgically excised in veterinary hospitals or private clinics in Portugal. A portion of each tissue sample was frozen for molecular analyses, while the remaining samples were fixed in 10% neutral buffered formalin for histopathological diagnosis. Sixteen adjacent mammary tissues were collected from animals harboring tumor samples. Two normal mammary gland tissue samples were obtained from two dogs undergoing postmortem examination. Age and breed were registered.

Histological sections (two µm-thick) were stained with hematoxylin and eosin (H&E) and analyzed under a light microscope with a digital camera by a trained veterinary pathologist (A.G.) (Nikon Eclipse E6000^®^, Nikon Instruments Inc., Melville, NY, USA). Tumor samples were classified histologically using the classification system proposed by the Davis-Thompson DVM Foundation [22]. The samples were divided into three groups: 18 control samples (16 tumor-adjacent and 4 normal mammary tissues), 6 benign samples (4 benign mammary tumors and 4 mammary hyperplasias), and 27 malignant samples (all malignant mammary carcinomas).

### 2.3. Total RNA Extraction

Tissue samples were processed for total RNA extraction using the semi-automated high-throughput magnetic-bead extraction kit (MagMAX™ mirVana™ RNA Isolation Kit; product no: A27828, Thermo Fisher Scientific^®^, Waltham, MA, USA) on the KingFisher Duo Prime magnetic particle processor (Thermo Fisher Scientific^®^, Waltham, MA, USA) following the manufacturer’s instructions.

RNA concentration and purity were analyzed using the NanoDrop Lite spectrophotometer (Thermo Fisher Scientific^®^, Waltham, MA, USA) and further stored at −80 °C until use.

### 2.4. cDNA Synthesis

RNA samples served as a template for cDNA synthesis using a TaqMan™ MicroRNA Reverse Transcription kit (Applied Biosystems^®^, Foster City, CA, USA) according to the manufacturer’s guidelines. The reverse transcription reactions were performed in MyCycler™ Thermal cycler (Bio-Rad Laboratories, Hercules, CA, USA) for 30 min at 16 °C, 60 min at 42 °C, 10 min at 85 °C, and then held at 4 °C, after which the cDNA was stored at −20 °C.

### 2.5. Relative Quantification of microRNAs

The miRNA expression levels were measured by quantitative real-time polymerase chain reaction (RT-qPCR) using the StepOnePlus^TM^ qPCR system (Applied Biosystems^®^, Foster City, CA, USA). Data analysis was performed using the StepOne software (version 2.3, Applied Biosystems^®^, Foster City, CA, USA).

The RT-qPCR reaction was performed for each sample in a final reaction volume of 10 µL, using 3.5 µL of nuclease-free water, 5 µL of 2× TaqMan^®^ Fast Advanced Master Mix (Applied Biosystems^®^, Foster City, CA, USA), 0.5 µL of 20× TaqMan^TM^ Gene Expression Assays (miR-29b: 000413, miR-125b: 000449, miR-146a: 000468, let-7b: 002619, mir-16: 000391, Applied Biosystems^®^, Foster City, CA, USA), and 0.5 µL of cDNA sample. miR-16 was selected as the endogenous control for normalization, as described previously [23,24,25,26]. The thermal cycling conditions were as follows: 10 min at 95 °C followed by 40 cycles of 15 s at 95 °C and 1 min at 60 °C. The quantification of miRNAs was performed in duplicate, and CT standard deviation values superior to 0.5 were excluded from the analysis. Negative controls (without cDNA) were also included in all reactions.

### 2.6. MicroRNAs Predicted Target and Enrichment Analysis

The potential biological implications of the studied miRNAs were explored through *in silico* analyses. Target genes prediction for let-7b, miR-29b, and miR-125b were performed using the online database miRWalk 3.0 [http://mirwalk.umm.uni-heidelberg.de (accessed on 24 May 2024)], which includes 3 miRNA-target prediction databases (miRDB, TargetScan and miRTarBase) [27]. The analysis targeted miRNA binding sites in the 5′ untranslated region (5′UTR), in the coding sequence (CDS), and the 3′UTR, selecting all potential targets with a binding probability of 1. To avoid redundancy, gene targets linked to more than one miRNA were removed from the final set.

These miRNA targets were then visualized in a protein–protein interaction (PPI) network using STRINGapp Protein Query from Cytoscape software (v3.10.2; https://cytoscape.org, accessed on 7 June 2024) with a confident score cut-off of 0.4. A Markov Cluster Algorithm (MCL) was further applied to cluster the proteins based on their STRING interaction score, with an inflation value of 3.0. Lastly, functional enrichment of formed clusters was performed using the canine genome as background. This analysis was performed using the enrichment analysis tool of the STRINGapp Protein Query, considering a False Discovery Rate (FDR) of *p* < 0.05. The enrichment results were filtered to remove redundant terms using a cut-off of 0.5. Gene Ontology (GO), Kyoto Encyclopedia of Genes and Genomes (KEGG), and Reactome terms were selected.

### 2.7. Statistical Analysis

Data analysis was performed using GraphPad Prism^®^ 9.5.0 software for Windows (GraphPad Software Inc., La Jolla, CA, USA). The Shapiro–Wilk or Kolmogorov–Smirnov test was applied to determine the data for normal distribution. Depending on the distribution, the Student’s *t*-test (for normal distribution) or Mann–Whitney U test (for non-normal distribution) was used to assess statistical differences between the expression levels of the normalized miRNAs. All values were considered statistically significant at *p* < 0.05. Pearson’s correlation coefficients were calculated for the gene expression data (-∆Ct values) of several of the microRNAs to identify any potential relationships among their expression levels.

## 3. Results

### 3.1. Clinicopathological Features

In this study, fifty-three mammary samples from 34 female dogs were included with a mean age of 8.25 ± 2.86 years (mean ± standard deviation), ranging from 4 to 15 years. The clinicopathological findings are summarized in Table 1. Most of the animals were mixed breeds (n = 12; 38.8%), followed by German Shepherds (n = 5; 16.1%), and the majority were less than 10 years old (n = 19; 67.9%). Malignant tumors were removed from female dogs aged 7.67 ± 1.37 years, while the benign samples were harvested from animals aged 9.05 ± 3.11 years. No significant statistical differences were observed in age between female dogs with malignant and benign mammary tumors.

The histological diagnosis of the canine mammary samples is detailed in Table 2. Benign tumors were diagnosed as complex adenomas (n = 2; 3.8%) and benign mixed tumors (n = 2; 3.8%) (Supplementary Figure S1). Malignant tumors were mostly classified as complex carcinomas (n = 12; 22.6%) and tubulopapillary carcinomas (n = 7; 13.2%).

### 3.2. miRNAs Expression Analysis

**Figure 1:**
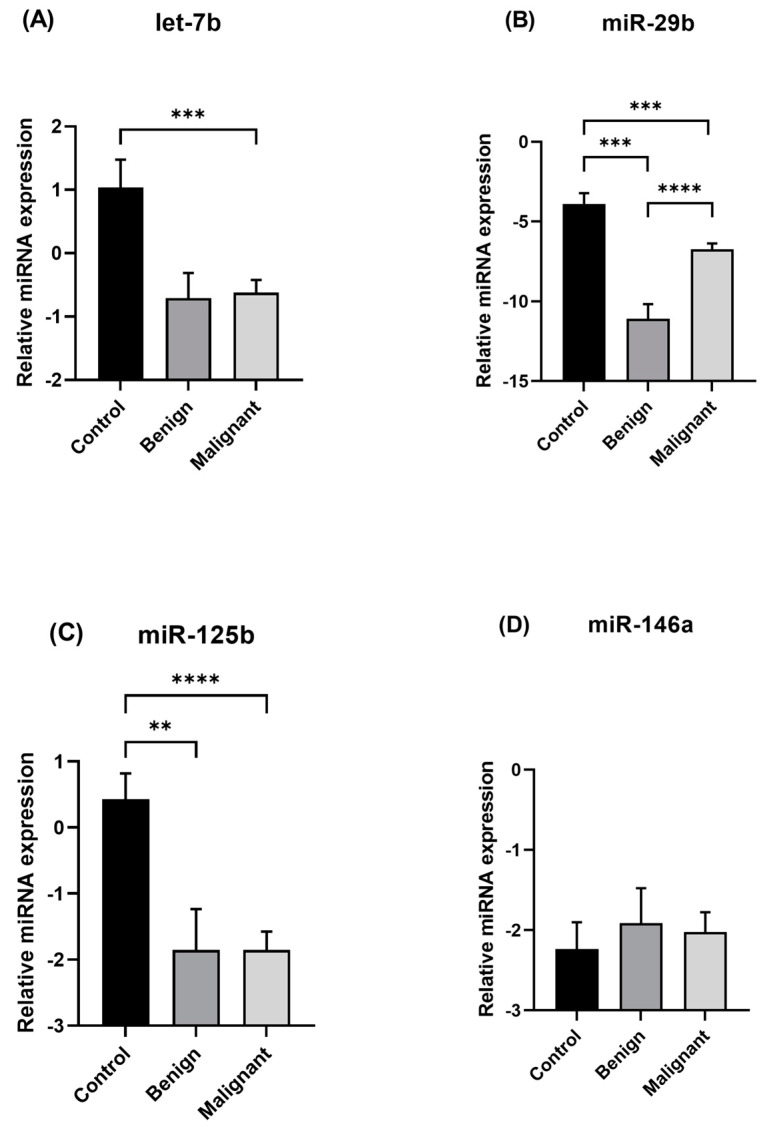
Relative expression levels of the miRNAs let-7b (**A**), miR-29b (**B**), miR-125b (**C**), and miR-146a (**D**) among benign lesions, malignant lesions, and the control group. Data are presented as −∆Ct mean values ± standard error of the mean (SEM). Statistical analysis was performed using Student’s *t*-test for normally distributed variables and Mann–Whitney U test for non-normally distributed variables. Significance was declared at *p* < 0.01 (**), *p* < 0.001 (***), and *p* < 0.0001 (****).

**Figure 2:**
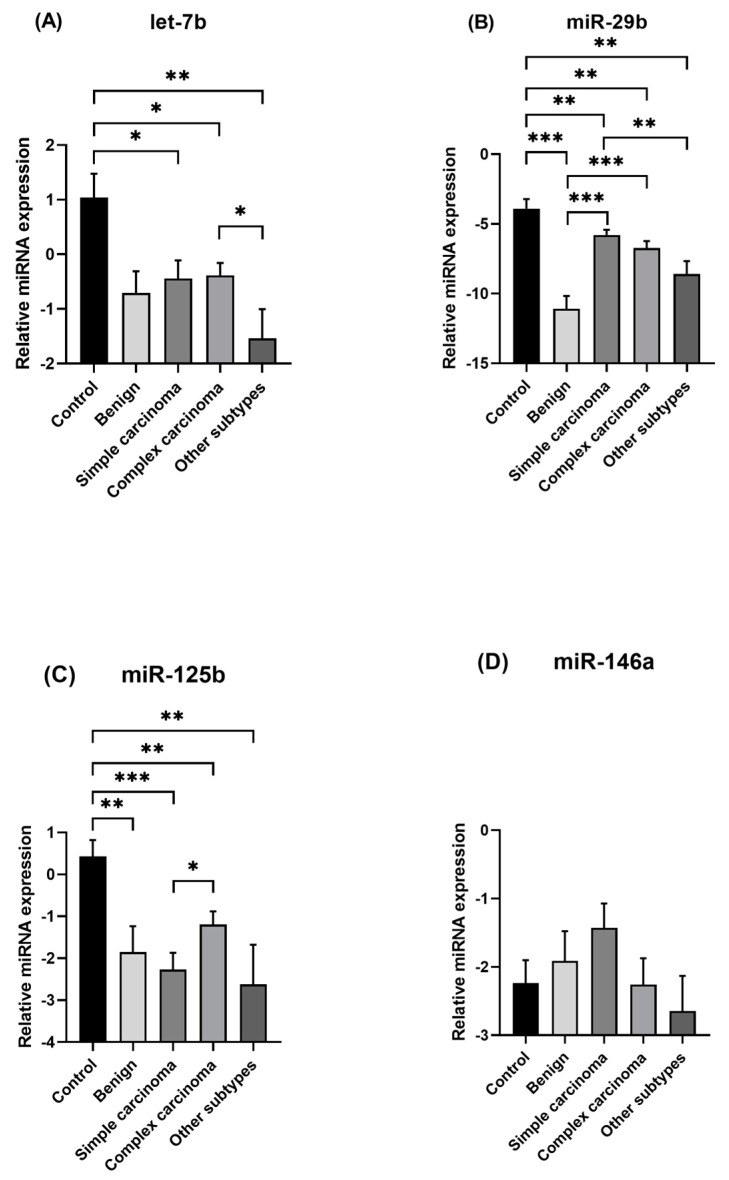
Relative expression levels of the miRNAs let-7b (**A**), miR-29b (**B**), miR-125b (**C**), and miR-146a (**D**) among benign lesions, simple, complex, and other subtypes carcinomas, and the control group. Data are presented as −∆Ct mean values ± standard error of the mean (SEM). Statistical analysis was performed using Student’s *t*-test for normally distributed variables and Mann–Whitney U test for non-normally distributed variables. Significance was declared at *p* < 0.05 (*), *p* < 0.01 (**), *p* < 0.001 (***).

Figure 1 displays the expression of miRNAs across the three groups: control, benign, and malignant lesions. Let-7b (*p* = 0.0004) was seen as downregulated in the malignant group when compared to the control group. miR-29b (*p* = 0.0001; *p* = 0.0003) and miR-125b (*p* = 0.0070; *p* < 0.0001) exhibited downregulation in both benign and malignant groups when compared to the control group. In addition, only miR-29b (*p* < 0.0001) showed significant differences between the benign and malignant groups, being upregulated in the malignant group. No significant differences were observed among the groups for miR-146a. Based on the two major histological types (simple and complex carcinoma), the malignant group was divided into simple carcinomas (including tubulopapillary, solid, and comedocarcinoma), complex carcinomas, and other carcinoma subtypes (carcinoma-and-malignant myoepithelioma and carcinosarcoma) for further comparison. Let-7b was significantly downregulated in simple carcinomas (*p* = 0.0295), complex carcinomas (*p* = 0.0189), and other carcinomas (*p* = 0.0085) compared to the control group (Figure 2). In addition, let-7b was significantly upregulated in complex carcinomas (*p* = 0.0313) in relation to other subtypes. MiR-29b exhibited a significant downregulation in all groups in comparison with the control group. Furthermore, miR-29b was upregulated in the simple carcinoma group (*p* = 0.0002) and complex carcinoma group (*p* = 0.0001) compared to benign cases. Moreover, miR-29b was significantly upregulated in simple carcinomas in relation to other subtypes (*p* = 0.0050). Regarding miR-125b, all groups were downregulated compared to the control group. In addition, in simple carcinomas, miR-125b was downregulated in comparison with the complex carcinoma group (*p* = 0.0437). No significant differences were observed among the groups for miR-146a.

Different degrees of positive correlation were found between all miRNAs, with significant correlation values observed between miR-29b and let-7b and (r = 0.5776, *p* < 0.001), miR-125b and let-7b (r = 0.8095, *p* < 0.001), and miR-125b and miR-29b (r = 0.5581, *p* < 0.001) (Supplementary Figure S2).

### 3.3. miRNA Target Prediction and Enrichment Analysis

**Figure 3:**
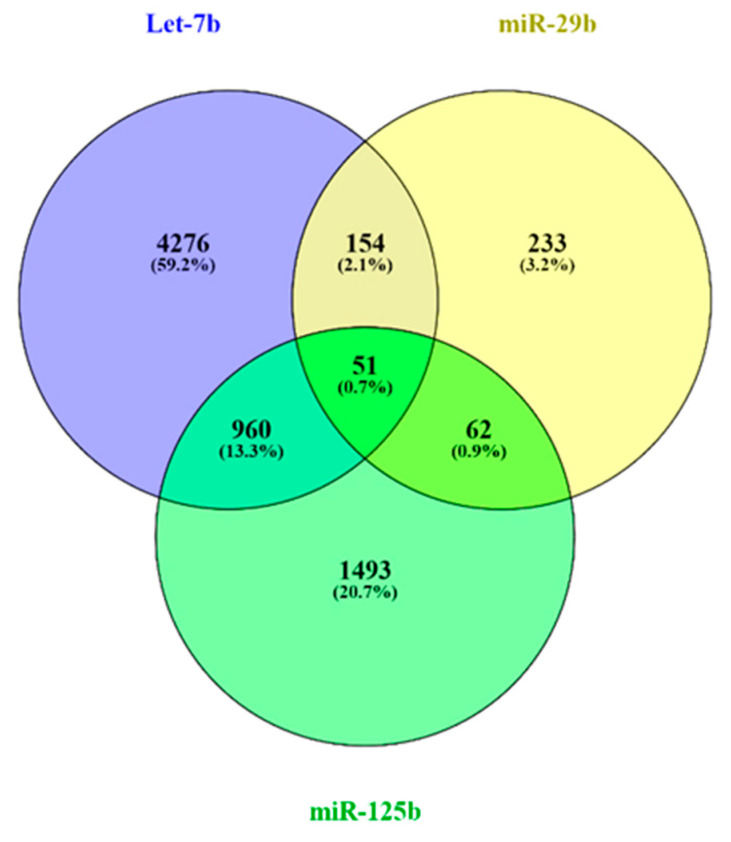
Venn diagram showing the number of unique and common target genes of let-7b, miR-29b, and miR-125b obtained from the miRWalk database, with a binding probability of 1. Graph obtained using Venny 2.1.

According to our results, let-7b, miR-29b, and miR-125b were the miRNAs found to be downregulated in the neoplastic lesions, suggesting that they may have interfered in tumor formation/progression. Thus, our *in silico* analysis focused on the mRNA targets of these 3 miRNAs. The miRNA–mRNA interaction resulted in a total of 7729 target genes (5441 for let-7b, 500 for miR-29b, and 2566 for miR-125b), of which 1227 were considered for STRING analysis due to their interaction with two or three miRNAs (Figure 3, Table S1). Fifty-one genes were found in common in the three miRNAs.

**Figure 4:**
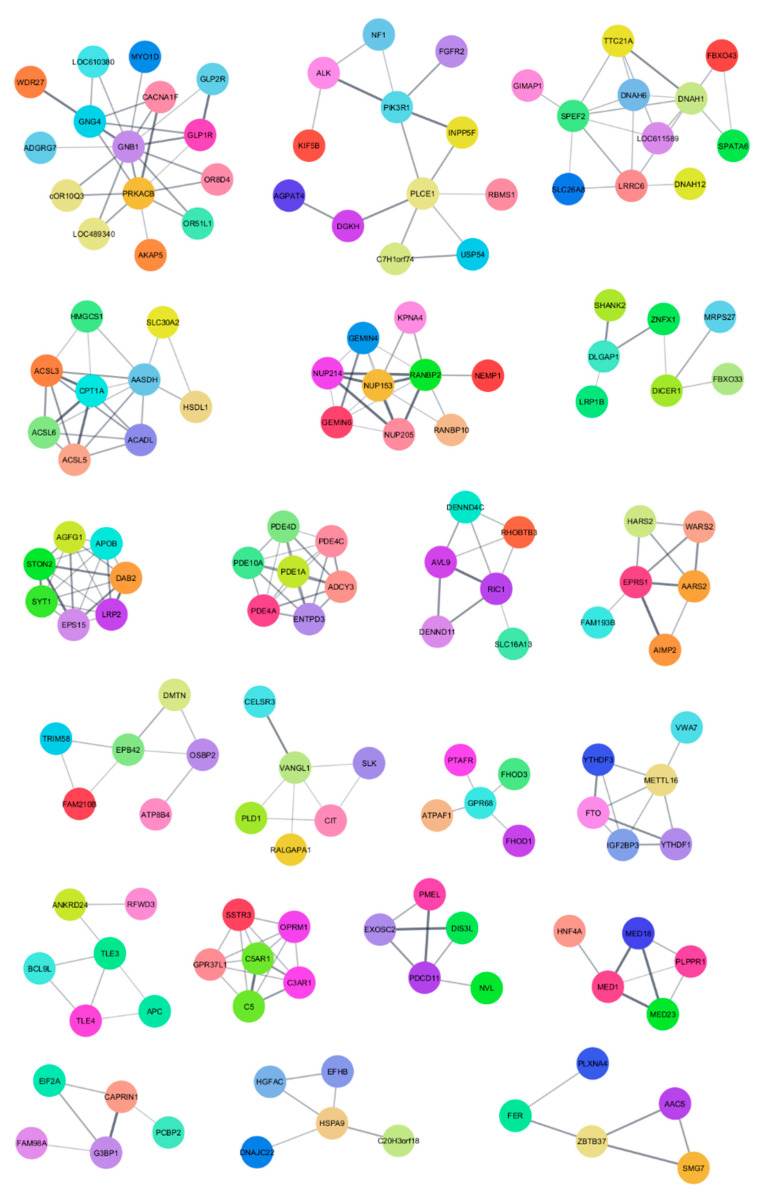
Protein–protein interaction (PPI) network of let-7b, miR-29b, and miR-125b. PPI was generated using the STRINGapp Protein Query from Cytoscape (v3.10.2), and clustering was performed by applying the Markov Cluster Algorithm (MCL), using an inflation value of 3.0. Proteins with fewer than five interaction partners in the network have been omitted from the visualization.

To analyze the biological impact of the three miRNAs, we analyzed the 1227 target genes using the STRINGapp Protein Query from Cytoscape (v3.10.2.) and by applying MCL. PPI networks were generated with a total of 1170 nodes and 629 edges, all with a significant enrichment (*p* = 1.0 × 10^−16^) (Figure 4).

**Figure 5:**
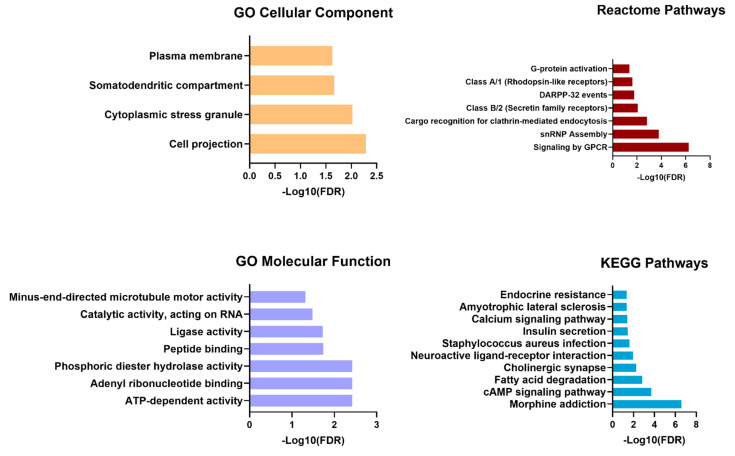
Gene ontology (GO), Kyoto Encyclopedia of Genes and Genomes (KEGG), and Reactome pathways of the functional enrichment analysis. The analysis was performed with STRINGapp Protein Query from Cytoscape (v3.10.2). Redundant terms were eliminated with a redundancy cut-off of 0.5, and an FDR threshold of 5% was applied.

For the functional enrichment analysis, we focused on clusters comprising five or more interactions, obtaining a total of 149 nodes with 243 edges across 21 clusters (Table S2). The analysis was conducted with an FDR threshold of 5%, and redundant terms were removed using a redundancy cut-off of 0.5, which resulted in a total of 28 functional enriched terms among the Reactome, KEGG, and GO categories (Figure 5, Tables S3–S6). Reactome pathways revealed that the targets of the deregulated miRNAs are involved in signaling by GPCR, whereas KEEG pathways demonstrated that miRNAs are involved in cAMP signaling and fatty acid degradation.

## 4. Discussion

This study aimed to understand the differences in the expression of four miRNAs (let-7b, miR-29b, miR-125b, and miR-146a) in dogs diagnosed with mammary tumors and to determine the diagnostic capabilities of single and combined miRNAs. The mean age of the female dogs included was similar to previous studies, which reported ages between 7 and 13 years [28,29,30,31]. Although not significant, more malignant tumors were found at younger ages than benign tumors (7.67 ± 1.37 vs. 9.05 ± 3.11 years), which is contradictory with other studies indicating that malignant neoplasm occurs in older dogs [32]. Our small cohort may be a reason for this result since it may not be large enough for epidemiological studies. The predisposition of mixed-breed dogs to develop CMTs was aligned with other studies [30,33,34]. However, this issue is controversial in literature and has been attributed to variations in the canine population included in the research studies [35]. Regarding histological classification, tubulopapillary and complex carcinomas were the most common subtypes, as previously described by several authors [29,30,36].

We selected benign and malignant lesions to assess whether there was a differential expression of miRNAs at different stages of canine mammary tumor progression. To analyze differential expression in specific cancer phenotypes, we selected homogeneous groups of tumors that were classified as simple (tubulopapillary, solid, and comedocarcinoma) and complex carcinomas. The remaining samples were grouped into a single group that included carcinoma-and-malignant myoepithelioma and carcinosarcoma.

A significant downregulation of let-7b was observed in malignant lesions compared to control cases. These results are in concordance with HBC, where it was inversely associated with lymph node metastasis, overall survival, and disease-free survival [18]. A recent study reported that the use of let-7b mimics in the MCF-7 cell line increased the expression of the pro-apoptotic BAX gene and decreased the expression level of anti-apoptotic BCL2 gene after let-7b overexpression, suggesting that this miRNA was associated with apoptosis-regulating genes in HBC [37]. Regarding the three histological subtype groups, upregulation was also found in complex carcinomas in relation to other subtypes. In our study, the downregulation of let-7b was not observed in benign lesions, which suggests that let-7b could be involved in the oncogenic process and differentiation of lesions. Contrary to our findings, let-7b and let-7b-5p were recently found upregulated in canine mammary cell lines and mammary tissues, respectively [38,39]. One possible explanation for this difference is that these studies used different endogenous microRNAs. Additionally, the cell lines were derived from a mixed tumor and a comedocarcinoma, which are not representative of our study. Furthermore, the study on mammary tissue samples did not stratify the data by malignancy status (benign vs. malignant lesions) or by histological type.

MiR-29b was shown to be downregulated in both benign and malignant lesions compared to control; however, malignant lesions were upregulated in relation to benign lesions. Our results are in concordance with studies in which miR-29b was downregulated in benign and malignant canine lesions compared to control [40,41]. In these studies, miR-29b showed no significant differences between benign and malignant lesions [40,41]. In our study, this difference was observed, demonstrating its ability to differentiate benign from malignant lesions. Curiously, after the analysis of miR-29b expression between the histological subtypes, our data showed an upregulation of two groups (simple and complex carcinomas) in relation to benign lesions. These results indeed demonstrate the complex role of this miRNA, which might have both tumor suppressor and oncogenic roles. Additionally, the expression of miR-29b was significantly increased in simple carcinomas compared to other subtypes. A study observed a downregulation of miR-29b in metastatic canine samples compared to the primary tumor [16]. In contrast, another study described the upregulation of miR-29b in canine mammary cancer samples compared to the control, but the authors used RNU6 as the reference gene for normalization, which could explain these differences [15]. Interestingly, in CMT cell lines, miR-29b was also found to be upregulated [42,43], as well as in exosomal RNA [26].

According to our results, miR-125b was shown to be downregulated in both benign and malignant lesions, as well as in the three groups (simple, complex, and other subtypes), compared to the control group. These findings suggest that miR-125b can be a diagnostic biomarker, confirming the presence of a neoplastic lesion. In addition, miR-125b seems to have a malignant differentiation capacity, since it can distinguish simple carcinomas from complex carcinomas. In another study, miR-125b was found to be downregulated in canine mammary neoplastic lesions [16]. Moreover, Von Deetzen and co-workers evaluated the same microRNA and found no significant difference between canine mammary tissues (n = 30) at different stages of tumor progression (adenoma, non-metastasizing carcinoma, metastasizing carcinoma, and metastasis) and normal gland that used miR-181b, miR-155, and U44 for normalization [41]. Accordingly, in HBC, several studies reported that miR-125b was found downregulated in tissue samples [19,44]. Contradictory, a recent study observed a higher expression level of this miRNA in the serum of 56 patients with non-responsive breast cancer, indicating that it was associated with chemoresistance [45]. Another study using paraffin-embedded breast cancer samples of 221 patients described a positive association between high expression of miR-125b and tumor size and stage in HER2-positive breast cancer patients [46].

Since let-7b can distinguish between complex and other subtypes, miR-29b between simple carcinomas and other subtypes, and miR-125b between simple and complex carcinomas, using these three miRNAs together could allow a precise diagnosis in distinguishing among these three histological types. In sum, the differential expression of let-7b, miR-29b, and miR-125b together may suggest the ability to discriminate these two histological groups (simple and complex carcinoma) from the other subtypes. However, further studies with other specific histological types will be necessary to consolidate these results.

Regarding the bioinformatic analysis, our key findings from Reactome pathways suggest that these miRNAs are involved in cancer-related key pathways, namely G Protein Coupled Receptor (GPCR) that are transmembrane signal transducers that regulate physiological and pathological processes represent the largest family of therapeutics targets [47]. As a member of the GPCRs family, protein-coupled estrogen receptor (GPER), is a seven-transmembrane receptor that mediates estrogen signals in both normal and malignant cells and has been involved in diverse signaling pathways that characterize the progression of breast cancer [48]. GPER expression was associated with tumor size, metastasis, and tamoxifen resistance [49]. It has also been observed that it is more prevalent in triple-negative breast cancer and is associated with younger age and more aggressive disease [50]. The other GPCR, GPR110, influences the growth of HER2-positive breast cancer cells and potentiates drug resistance in these cells [51]. In the KEGG pathway, other cancer-related pathways were observed, including the cAMP pathway and fatty acid degradation, which also plays an important role in the development of HBC [52,53]. Previous studies have demonstrated that activation of the cAMP pathway can inhibit cell proliferation and migration in breast cancer [54,55]. Deregulation of fatty acid metabolism is recognized as a critical factor for malignant transformation in HBC and has been implicated in the promotion of cell proliferation, migration, and invasion, as well as associated with poor prognosis in breast cancer patients [53,56,57,58].

This study could be a breakthrough in the research of complex carcinoma (malignant adenomyoepithelioma) in HBC. Complex carcinoma is characterized by the proliferation of both luminal and myoepithelial cells, a rare condition in HBC. However, this type of tumor shows molecular similarities to those found in humans, making canine complex carcinoma a unique model for investigating the roles of myoepithelial cells, the second major cell lineage in the mammary gland involved in normal developmental and pathogenic processes [59].

Our study’s limitations include the use of tumor-adjacent mammary tissues as controls and only two normal mammary glands. In HBC, healthy control tissue is generally readily available through breast reduction surgeries [60]. However, this is not the case for canine patients, as mammary surgeries are not performed in veterinary clinics unless there is an existing pathology. Another limitation is the *in silico* analysis, which, although helpful in identifying biological mechanisms associated with these miRNAs, requires validation to provide an integrated view of adjacent mechanisms, as other genes and pathways with significant roles may have been overlooked. However, this issue was mitigated by the fact that the analysis was conducted only on genes whose mRNAs were regulated by at least two of the miRNAs studied.

Further studies are required to understand the mechanisms of these miRNAs and to consider them as potential biomarkers in dogs. Studies with larger sample sizes, using exclusively normal mammary gland tissue as controls, and investigating miRNAs not only in different histological types but also with diverse immunophenotype subtypes, could provide valuable insights. In HBC, miRNAs have been shown to exhibit distinct regulation across immunophenotype subtypes [61]. It is also necessary to associate these miRNAs with clinicopathological features, such as tumor histological grade, tumor size, and lymphatic invasion, for these miRNAs to be used as prognostic biomarkers. The use of animal samples with follow-up will also facilitate a better understanding of the prognostic value of these miRNAs. In addition, functional assays will allow us to understand the involvement of these miRNAs in tumorigenesis, including their roles in proliferation, apoptosis, migration, and invasion in cell culture models, as well as in the evaluation of tumorigenicity and metastasis in xenograft animal models. Additionally, the use of antimiRNAs or miRNAs mimics in both *in vivo* and *in vitro* models would further help to consolidate the role of these miRNAs in tumorigenesis. Finally, incorporating these miRNAs into non-invasive techniques, such as serum or exosomal miRNA profiling, could improve the early detection and prognosis of CMTs, thereby increasing their translational relevance and potential clinical applications.

## 5. Conclusions

Histological classification guides diagnostic and prognostic evaluation, suggesting that the differential expression of miRNAs across different histological types is a promising line of research. The expression of miR-29b differed significantly between benign and malignant groups, indicating its potential to differentiate malignancy, while let-7b, miR-29b, and miR-125b appear capable of discriminating between histological phenotypes. Further studies with these miRNAs and their combined use could improve diagnostic accuracy.

This work establishes a miRNA signature for CMT to guide future miRNA selection for early validation and reinforces the evidence supporting microRNAs as important biomarker candidates for this tumor type in dogs.

## Figures and Tables

**Table 1:** Clinicopathological findings of female dogs included in this study.

Features	Frequency (%)
**Age (n = 28)**	
>10 years	9 (32.1%)
≤10 years	19 (67.9%)
**Breed (n = 31)**	
Mixed	12 (38.8%)
German shepherd	5 (16.1%)
Boxer	4 (12.9%)
Pekingese	2 (6.5%)
Portuguese Pointer	2 (6.5%)
Cocker Spaniel	1 (3.2%)
Dachshund	1 (3.2%)
French bulldog	1 (3.2%)
Labrador Retriever	1 (3.2%)
Pinscher	1 (3.2%)
Poodle	1 (3.2%)

**Table 2:** Distribution of canine mammary samples according to histological classification.

Histopathological Group	Histological Classification	Frequency (%)
**Controls (n = 18)**	Normal mammary gland	2 (11.1%)
	Adjacent mammary gland	16 (88.9%)
**Benign lesions (n = 6)**	Lobular hyperplasia	2 (33.3%)
	Complex adenoma	2 (33.3%)
	Benign mixed tumor	2 (33.3%)
**Malignant lesions (n = 27)**	Tubulopapillary carcinoma	7 (25.9%)
	Solid carcinoma	2 (7.4%)
	Comedocarcinoma	1 (3.7%)
	Complex carcinoma	12 (44.5%)
	Carcinoma-and-malignant myoepithelioma	2 (7.4%)
	Carcinosarcoma	3 (11.1%)

## Data Availability

The data are contained in the manuscript and Supplementary Materials.

## Supplementary Materials

The following supporting information can be downloaded at: https://www.mdpi.com/article/10.3390/ani15010020/s1, Figure S1. Histopathology microphotographs representative of the canine mammary gland tissues analyzed in the present study: controls (A. normal mammary gland); benign lesions (B. complex adenoma); malignant lesions (C.tubulopapillary carcinoma and D. complex carcinoma). Hematoxylin and eosin staining. Figure S2. Heatmap of Pearson correlation coefficient matrix. The correlation matrix heatmap displays the Pearson correlation coefficients between microRNA expression data. In this matrix, 1 indicates a perfect positive correlation, 0 indicates no correlation and −1 indicates a perfect negative correlation between the studied microRNAs. Statistical significance: * *p* < 0.05, ** *p* < 0.01, *** *p* < 0.001, **** *p* < 0.0001. Table S1. List of 1227 gene targets obtained from the mRNA/miRNA interaction, selected for STRINGapp Protein Query. Table S2. Protein-protein-interaction (PPI) network clusters according to Marckov Clusterring (MCL) analysis. Only clusters with five or more interactions were considered. Table S3. Gene Ontology (GO) Cellular Component enrichment analysis for terms with FDR *p*-value < 0.05. Table S4. Gene Ontology (GO) Molecular Function enrichment analysis for terms with FDR *p*-value < 0.05. Table S5. KEGG enrichment analysis for terms with FDR *p*-value < 0.05. Table S6. Reactome pathway enrichment analysis for terms with FDR *p*-value < 0.05.
