# Supplementary material for: Let-7b, miR-29b, and miR-125b as Potential Biomarkers for Differentiating Canine Mammary Carcinoma Histological Types
Source: Animals (Basel). 2024 Dec 25;15(1):20. doi: 10.3390/ani15010020 (PMC11718878; doi:10.3390/ani15010020)
Supplement: Supplementary file 1 [file animals-15-00020-s001.zip › animals-3363821-supplementary.pdf]

animals

Let-7b, miR-29b and miR-125b as potential biomarkers for differentiating canine mammary carcinoma histological types

Supplementary Material

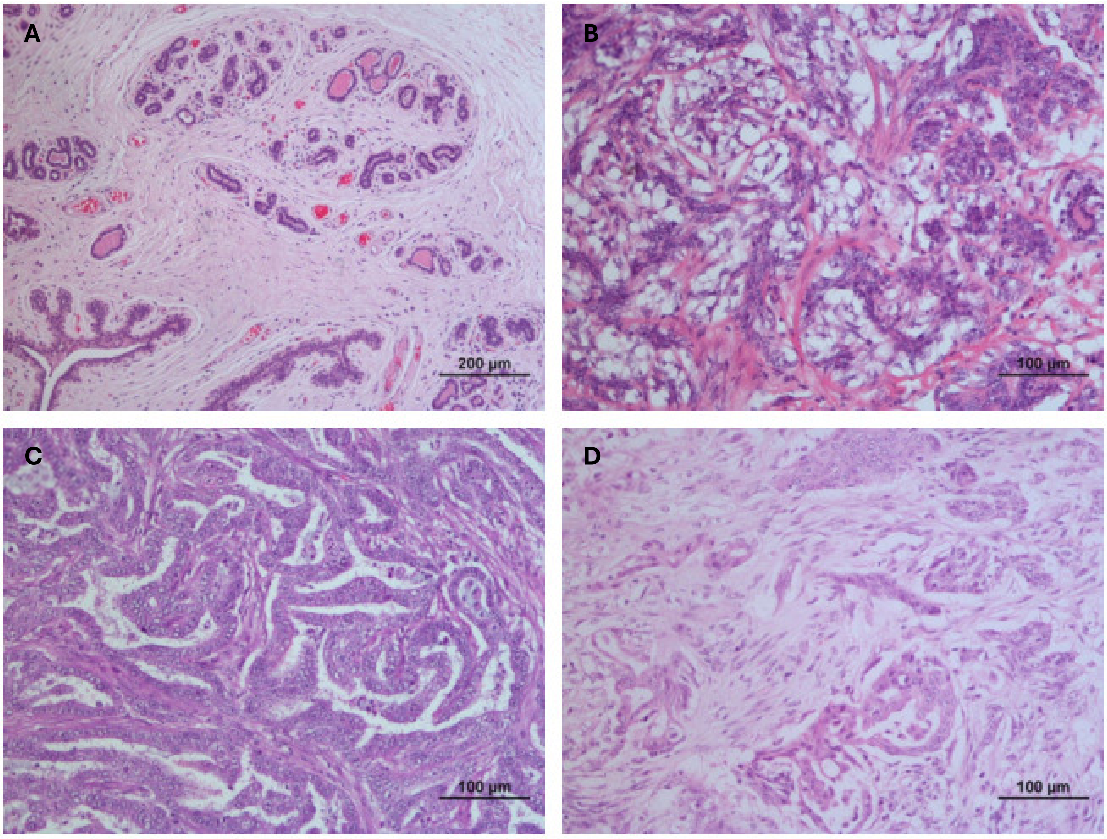

Figure S1. Histopathology microphotographs representative of the canine mammary gland tissues analyzed in the present study: controls (**A.** normal mammary gland); benign lesions (**B.** complex adenoma); malignant lesions (**C.** tubulopapillary carcinoma and **D.** complex carcinoma). Hematoxylin and eosin staining.

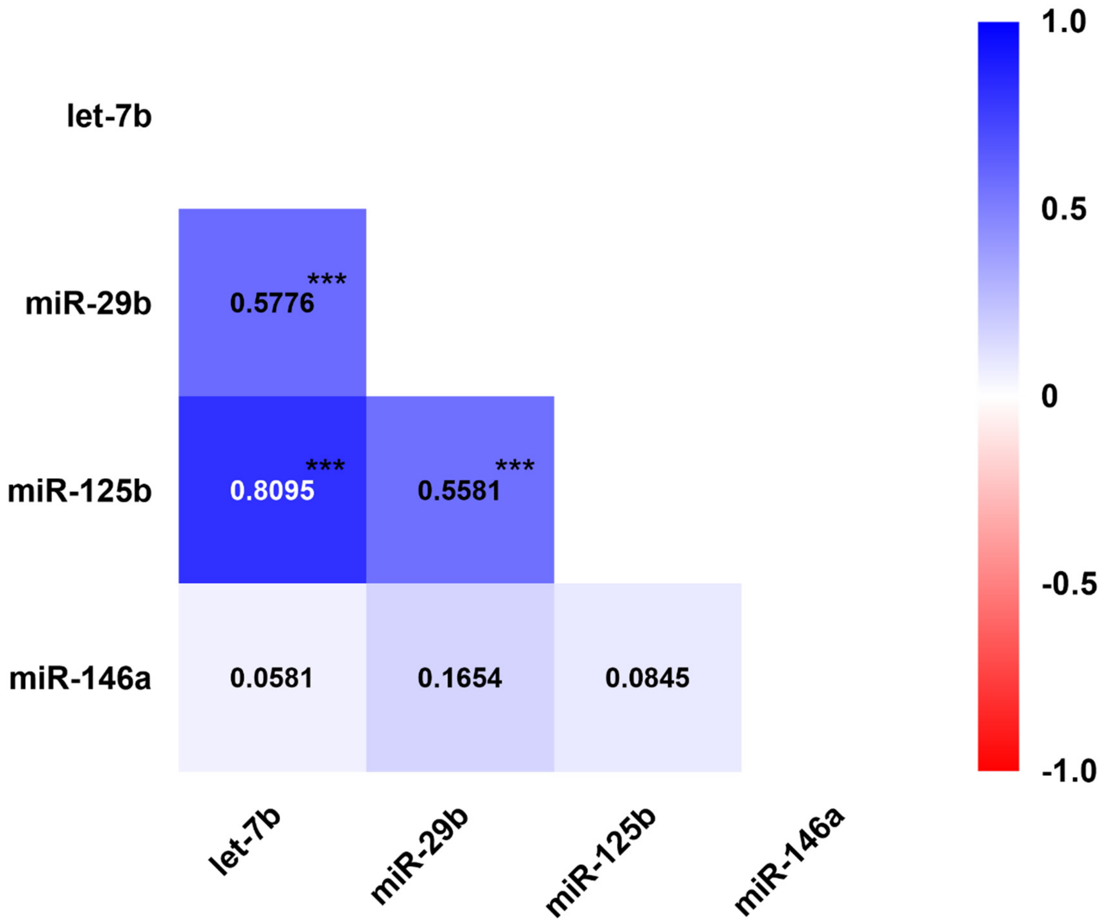

Figure S2. Heatmap of Pearson correlation coefficient matrix. The correlation matrix heatmap displays the Pearson correlation coefficients between microRNA expression data. In this matrix, 1 indicates a perfect positive correlation, 0 indicates no correlation and −1 indicates a perfect negative correlation between the studied microRNAs. Statistical significance: * $p<0.05$, ** $p<0.01$, *** $p<0.001$, **** $p<0.0001$.

Table S1. List of 1227 gene targets obtained from the mRNA/miRNA interaction, selected for STRINGapp Protein Query.

microRNA	Gene target
let-7b, miR-29b and miR-125b	*RALGAPA2, ZNF641, NFASC, AGTPBP1, BCL9L, WDR59, PRRC2C, SYT16, CBX6, ZNF451, GTF3C6, FGD6, FBLN7, DUSP16, ISL1, OXR1, DBH, ATM, ZDHHC5, DEAF1, PLK4, MITF, DNAH1, CELSR3, OTOG, CLASP2, GORASP1, TTC21A, CIT, TACC2, PXDNL, VGLL3, LRP2, SYNE1, CIC, VPS13D, CHD1, RHOBTB3, FUT11, NCOR1, WIPI2, PARP1, TOP2A, LCN10, LRRC8A, SMC1B, DNAH8, IRF5, AASS, NRG1, KIDINS220*
let-7b and miR-29b	*GABPB2, TCP11L1, SLC7A11, CCDC93, LOC119864502, EIF4E3, KBTBD8, RBM15B, ANKRD49, CLYBL, ACP3, FAM210B, BICD1, CCND2, RBM20, MAP1A, RFX7, BNIP2, LOC478384, FAM13A, DNAH5, MYNN, LOC100685048, ZC4H2, RLIM, ZNF516, SPIN1, CUL2, TRIM23, VWA5B1, PGD, NTRK3, MPZL3, SLC16A13, SLC35D1, SUN1, LOC119872207, DTL, FAM163A, ZBTB37, POU2F1, PLD5, CNST, SGPP1, CDC42BPB, NRXN1, FEM1C, HSPA9, SVEP1, KIAA1958, GJA9, PTPRQ, VANGL1, CSTF3, GJB6, NEXMIF, CD226, RFX3, PROM1, ARRDC3, ZDHHC4, RABGAP1L, PDE6B, ABCC1, COL1A1, GNB1, TRPV4, TES, ATP2B1, OPRM1, OR51L1, ATXN7L1, PCDH18, INPP4B, LRP1B, DNAH12, SYDE1, MYO7A, FAM160A2, E2F8, LPAR6, LOC119865171, ENTPD3, ATR, CPXM1, UGT1A6, PDS5B, DOCK10, CUX2, CNTN1, WNK1, FGFR2, GPR176, FAM214A, ACAP2, ATP13A3, ACTRT3, ZNF639, PRKAG3, IKBKE, EPRS1, CACNA1F, ERCC6L, NAA10, SPRY3, AHI1, LAMA2, TLE4, LOC476416, WAC, DHTKD1, FTO, UBR4, C2H10orf67, MACIR, MCTP1, LYSMD4, USP54, RGR, FCSK, PHLPP2, FHOD1, BAZ1B, ATP5J2, AIMP2, DOC2A, WDR64, CEP76, ZFHX2, NEK9, KCNK10, DHX8, DUSP14, PMPCA, RC3H2, PLAA, AARS2, MLIP, ASCC3, LRRC6, LNX1, AASDH, POLR2B, LOC482174, CALU, ANKIB1, MIOS, SP4, EPS15, ATPAF1, CNOT4, AGBL3, SH3BP5L, LOC119874670*
miR-29b and miR-125b	*EIF4E2, SLC1A2, PGRMC2, PGPEP1, YTHDF1, DCLK1, PATZ1, DENND5B, PARP11, BCL2L13, DAPK2, ARHGAP31, AMER1, SGK1, ASPN, SUV39H2, PURA, PI4K2B, RERE, IST1, SNX8, GGA2, IGF2BP3, ZNF862, HIPK1, HTD2, FBXO43, TRPV1, UGCG, EHBP1L1, PGLYRP2, EED, DLG2, KIAA1671, SLC38A2, INO80, HERC3, ANK2, DSP, ZKSCAN4, TLK1, ATP7A, PEG3, KLC3, PARP8, PATJ, TTC34, PSKH1, ELMO3, BRCA1, MYO1C, POLR3B, ECPAS, C5, SCIN, CRHR2, HIVEP3, TMEM154, DENND11, FAM98A, ARID5A, TENT5C*
let-7b and miR-125b	*CLCN1, C5AR1, NKX2-5, CGN, SLC23A2, TXNRD1, PIM1, COL9A3, MAPK3, CPT1A, PTAFR, NECTIN1, PDS5A, DSC1, POLR3GL, MINDY1, VSTM2A, HIPK3, CELF1, MADD, CREB3L1, TP53I11, SHANK2, CTSF, BBS1, NAA15, TPRA1, EFCC1, TMEM43, GRIP2, NR2C2, RFT1, PCBP4, ALS2CL, LOC484867, PTGER1, TNPO2, PDE4A, BSG, RNF141, PAK1, B3GNT6, SLCO2B1, FAM168A, LOC106560171, TRIM66, UEVLD, GPALPP1, GRK1, CRTAP, LOC607986, MYRIP, XRN1, KCNAB1, GZF1, RBBP9, GPCPD1, TPX2, PLAGL2, PPP1R16B, HNF4A, RIMS4, ELMO2, SLC7A1, SLC25A15, FBXO16, DMTN, ZNF605, TMEM132B, HECTD4,*

CFAP73, MED13L, FBXO21, KSR2, MLEC, OSBP2, CLTCL1, PPFIBP1, TESPA1, SPRYD3, DNAJC22, FGD4, C27H12orf60, EMP1, STYK1, GABARAPL1, C3AR1, PLCE1, TNKS2, LOXL4, HIF1AN, POLL, SLK, HABP2, ATE1, ASPH, FAM110B, NSMAF, PAG1, RBM12B, RAB27A, FMN1, ZNF106, MYEF2, CGNL1, ZNF609, NPTN, EDC3, HMG20A, ITSN1, B3GALT5, FRAS1, PDLIM5, CXXC4, PLA2G12A, NSUN3, ZPLD1, USF3, ECE2, LIPH, MASP1, KPNA4, PLD1, BTN1A1, ZKSCAN8, PDE1A, ABCB11, COBLL1, DCAF17, MAP3K20, SESTD1, HECW2, PPIL3, CDK15, NBEAL1, CREB1, PLEKHM3, ACADL, LMX1A, LOC609484, LOC478984, LOC612524, FANCB, SUV39H1, SMC1A, XKRX, TBC1D8B, LOC102157101, IRS4, TMEM164, LONRF3, LOC492092, NKAP, ZBTB33, ATP1B4, HMGB3, TBPL1, SCN1B, KLK13, ENPP1, ELAC1, UST, SOD2, PRKN, WDR27, PRUNE2, GCNT1, APBA1, TMEM252, S1PR3, BICD2, PRKCG, LOC484343, IGLON5, GYS1, ZNF529, LOC119863924, PLXDC2, KIF5B, FRMD4A, ECSCR, HBEGF, ZMAT2, KCTD16, STK32A, PLPP1, MAP3K1, CDK7, ZNF366, SERINC2, MED18, SLC30A2, WNT4, MTOR, TMC3, EFNA5, MTX3, LHFPL2, NIPA1, APBA2, SV2B, ABHD2, FANCI, ALPK3, HOMER2, RGS12, MSANTD1, EVC, TBC1D19, LGALS8, SPEF2, ZNF248, GNG4, EIF4EBP2, ERGIC1, KCNIP1, WWC1, LSM11, G3BP1, DAB2, NCAM1, RNF222, GLP2R, NLRX1, HYOU1, PAFAH1B2, GAS7, AKAP10, LOC489534, NATD1, SAMD11, ATAD3A, ANKRD65, MVD, ZNF469, LOC119863920, KLHL36, HSD17B2, RFWD3, ST3GAL2, KCNA2, BCL7B, SSC4D, KDELR2, LOC489893, MAD1L1, GPR146, STX1B, RNF40, ZNF48, ZKSCAN2, PRKCB, SYT17, RBFOX1, TBC1D24, PRKACB, TNFSF4, NAV1, DYRK3, C7H1orf74, TATDN3, ODR4, TNR, C7H1orf112, HCN3, UBE2Q1, ST8SIA5, C7H18orf25, GAREM1, COLEC12, CIDEA, TC2N, EFS, NOVA1, MAPK1IP1L, ESR2, AKAP5, GALNT16, SUSD6, MAP3K9, LIN52, CIPC, TBC1D16, C1QTNF1, AFMID, GPRC5C, CRHR1, CD300LG, KRT39, GSDMA, PSMD11, ADAP2, RAB11FIP4, NF1, NXN, INPP5K, METTL16, RAPGEF1, ABL1, EXOSC2, OLFML2A, NEMP1, SPRYD4, OS9, HMGA2, KIAA0930, TAB1, TCP11L2, TMEM182, CNNM4, MAML1, ZNF454, SLC22A5, JADE2, NFIB, SLC24A2, UBE2R2, PIGO, SIT1, PAX5, PLPPR1, TAL2, ZNF483, RGS3, LOC100856137, SLC26A8, GLP1R, TTBK1, TRAM2, SIM1, QRSL1, LOC482176, TRHR, LIMCH1, TMEM33, SLC30A9, CLOCK, PLEKHA8, NEUROD6, PRSS38, PLXNA4, AGR2, ITGB8, RAPGEF5, HOXA13, TFEC, NT5C1A, GRIK3, KIAA0319L, MOB3C, RNF220, TIE1, SYT1, TCAF1, KDM7A, DGKI, GPAT4, UNC5D, CFAP97, STOX2, SPCS3, CENPO, DNAJC27, ASXL2, HADHA, CRIM1, GEMIN6, ATOH8, PAIP2B, CSDE1, WARS2, VNN1, MGAT4F, MTMR11, EHF, LRRC55, INTU, ANKRD24, CFAP97D2, C24H20orf96, SLA2, FLT3, PRSS56, PISD, MARCHF8, CALHM2, ACSL5, SMIM34A, TIGD2, ERAS, DRP2, AKT2, NHSL1, AGPAT4, CALHM5, ZSCAN22, SSC5D, ZFP14, GRXCR2, PDE4D, PIK3R1, ZNF683, FER, FAM193B, PLEKHN1, PRKCZ, HSDL1, OSGIN1, PCOLCE, ITGAM, ANKS4B, LOC610677, NVL, LOC490399, RIPK3, CCDC57, P2RX1, IL17REL, APOM, GRHL2, COL14A1, MYCL, TMEM139, LOC100688918, ANK1, UMOD, PGB1, KCNJ2, TJP1,

CUBN, PON2, CYP2E1, CD34, PDGFRB, CCL8, LOC448801, CCKAR, ATP7B, HGFAC, KDM5C, ARSL, AOX2, CD47, SSPO, LOC100049001, PMEL, ACAN, OAZ1, FMC1, ANPEP, SGK3, P2RY1, TPC3, PRF1, VDAC3, STK38L, GMPS, CASQ2, NOS2, CAPRIN1, LOC106557476, TAC3, OR6B1, OR8S9, OR10G9, OR2W11, OR3A1H, OR51H5, OR52E22, OR8S12, OR8D4, PDE4DIPP2, ANKRD35, OTUD7B, APH1A, ANP32E, C17H1orf54, ADAMTSL4, POGZ, OR10V10, HECW1, MAGI2, UPP1, LRRC4C, QSER1, RIN1, EFEMP2, AP5B1, LOC476047, LOC119864445, IWS1, MAP3K2, LYPD6, RIF1, LOC119864504, IQCF1, SLC41A3, MKRN2OS, IRAK2, RPUSD3, GRM7, CHL1, ADAMTS9, ATXN7, CFAP20DC, ITIH4, STAB1, C20H3orf18, MST1, USP4, SETD2, LRRC2, SLC6A20, PDE4C, TMEM38A, CHERP, CYP4F22, LOC484960, INSR, ZNF358, DUS3L, DPP9, REXO1, RNF126, MMP26, SLC6A5, MTMR2, AMOTL1, USP35, POLD3, LOC476816, LAMTOR1, DCHS1, CYB5R2, C21H11orf16, NRIP3, PLEKHA7, LGR4, DGKH, CHAMP1, STAC, MLH1, TGFBR2, EFHB, PIK3R4, ZBTB38, PAQR9, EIF2A, IGSF10, BPIFB2, TMC2, KIF16B, JAG1, SLC52A3, ITCH, NCOA6, GGT7, NCOA5, ZNFX1, PRPF6, ACKR3, B3GLCT, PROSER1, NEK1, LOC486076, MTMR9, ESCO2, DPYSL2, CHMP7, XPO7, AGFG1, ATP6V0A2, SETD1B, TRAFD1, CABP1, SART3, MORC2, UFD1, CDKN1B, LOC486582, GPR84, HOXC6, PCBP2, DAZAP2, CSRNP2, TFCP2, ASIC1, KCNH3, DDN, LOC119866377, SOX5, GPRC5A, CLEC12A, SCNN1A, WASHC1, LBX1, TMEM273, RASGEF1A, BTAF1, HPS1, PKD2L1, SEMA4G, PPRC1, ARL3, WBP1L, PDCD11, PDZD8, INPP5F, EDRF1, ADAM12, RB1CC1, YTHDF3, NCOA2, PRTG, DISP2, MGA, SPTBN5, GANC, STARD9, TMEM62, EPB42, ATP8B4, HERC1, DPP8, DIS3L, TLE3, C30H15orf39, USP25, TIAM1, IFNAR2, IFNGR2, SON, GK2, WDFY3, AFF1, CPOX, POLQ, SLC15A2, MYLK, CCDC14, ITGB5, WDR53, ADGRG7, LPCAT1, EHHADH, LOC478670, LOC102153616, GCNT2, MAK, NUP153, LOC100855640, PJVK, RBMS1, HOXD10, HOXD4, TRAK2, MAP2, UNC80, ABCB6, ACSL3, MPZ, KLHDC8A, CDK18, MARK1, MIA3, ATF6, ATP1A4, USH2A, LOC611589, FRMPD4, CASK, BRWD3, PWWP3B, RADX, AMOT, XPNPEP2, LOC100688437, SECISBP2, LOC612523, ATP9B, SALL3, CDH19, SERPINB13, POLI, STXBP5, SASH1, SCAF8, ARID1B, FNDC1, PDE10A, TBC1D32, PTPRK, CDC14B, RIC1, ZNF865, TMC4, LOC102152131, LOC119881386, LOC100686511, LOC484365, PRMT1, SPHK2, HIF3A, NOVA2, FOXA3, TOMM40, WDR87, LRFN3, ZNF599, SVIL, TAF3, SPATA24, HARS2, PCDHB1, TCERG1, RAB3C, SLC30A5, MRPS27, CNOT1, RSPRY1, ABCC11, ZSCAN20, TRIM62, SPOCD1, GPATCH3, UBXN11, MAN1C1, EIF4G3, ARHGEF10L, TRPM1, SLCO3A1, SH2D7, APC, VCAN, FAN1, VPS33B, EFL1, NWD2, CCDC149, ADO, NRBF2, UNC5B, SEC24C, ANXA11, CLINT1, CYFIP2, HMGCS1, NIPBL, LMBRD2, FLI1, ETS1, DCPS, RPUSD4, CDON, TMEM123, NLRP1, ENO3, ACAP1, MPDU1, DNAH9, SLC47A2, MFAP4, TOP3A, MXRA8, LOC489640, DPEP1, SPG7, PIEZO1, CNTNAP4, FA2H, TLE7, RANBP10, UPK3BL2, DNASE2B, CALN1, TRIM56, SLC12A9, AGFG2, LOC119875773, SMURF1, CARD11, TAOK2, MARF1,

	LOC111096475, PDPK1, TEDC2, RAB26, TSC2, PIGQ, STXBP3, BCAR3, TGFBR3, TNNI3K, LRRC7, F13B, EMILIN2, GPR37L1, ZNF281, CRB1, UTP25, INTS7, CENPF, CEP350, SMG7, RASAL2, DNM3, MSTO1, CREB3L4, EPG5, FHOD3, TAF4B, TMEM241, SMCHD1, DLGAP1, RALGAPA1, FBXO33, KLHL28, ZFYVE26, DCAF5, STON2, GTF2A1, FLRT2, EML5, GPR68, DICER1, SYNE3, DYNC1H1, GRIN1, FZD2, P4HB, TNRC6C, NT5C, TMEM104, TANC2, SMARCD2, GPATCH8, STAT5A, STAT5B, ODAD4, LOC490972, MSL1, CDK12, MED1, HOXB3, LRRC59, TRIM25, BCAS3, MYO19, MYO1D, SUZ12, NLK, GEMIN4, SGSM2, CACNA1B, PNPLA7, GTF3C4, NUP214, FIBCD1, CERCAM, GAPVD1, SSTR3, CACNA1I, TIMELESS, TSPAN31, LRIG3, SRGAP1, TBK1, TCF20, ZC3H7B, ELFN2, BTBD11, RANBP2, MRPS9, PPP4R3B, USP34, ZNF608, ALDH7A1, PRRC1, ACSL6, FSTL4, BNC2, DENND4C, FANCG, UNC13B, MELK, ZBTB5, TRIM32, RPP21, CCHCR1, MED23, VWA7, LOC474850, C2, LOC481722, DXO, PPT2, PHF1, PKHD1, SMPD2, HS3ST5, RECQL4, PKHD1L1, SLAIN2, ADGRL3, EREG, LOC482200, TRIM58, CPA2, KLHDC10, HDAC9, ADCYAP1R1, AVL9, ANLN, KCND2, ADPRS, 37104, OSBPL9, AGBL4, SPATA6, MUTYH, NAV3, CEP290, UHRF1BP1L, MAB21L2, C15H4orf45, RAPGEF2, TLL1, LOC482707, LOC482755, SLC37A3, UBN2, NUP205, KRBA1, GIMAP1, AGAP3, SFRP1, LOC607729, DUSP26, PDLIM3, CAPN14, TCHHL1, PRR9, BIRC6, GREB1, LPIN1, APOB, ADCY3, TMEM214, SLC35F6, TRIM54, IFT172, ALK, LCLAT1, LOC100685565, GPAT2, ACOXL, LOC111090401, DNAH6, LOXL3, ZNF638

Table S2. Protein-protein-interaction (PPI) network clusters according to Marckov Clusterring (MCL) analysis. Only clusters with five or more interactions were considered.

Cluster	Clustered proteins
Cluster 1	*WDR27, AKAP5, PRKACB, cOR10Q3, LOC489340, OR51L1, LOC610380, GNG4, GLP2R, ADGRG7, MYO1D, GNB1, GLP1R, OR8D4, CACNA1F*
Cluster 2	*KIF5B, INPP5F, PLCE1, C7H1orf74, PIK3R1, USP54, NF1, AGPAT4, FGFR2, DGKH, ALK, RBMS1*
Cluster 3	*FBXO43, TTC21A, DNAH12, DNAH1, SPEF2, SPATA6, DNAH6, SLC26A8, LOC611589, GIMAP1, LRRC6*
Cluster 4	*ACSL5, ACSL3, HSDL1, SLC30A2, ACSL6, HMGCS1, CPT1A, AASDH, ACADL*
Cluster 5	*RANBP10, NUP153, RANBP2, GEMIN4, NUP214, KPNA4, GEMIN6, NUP205, NEMP1*
Cluster 6	*SHANK2, DICER1, FBXO33, ZNFX1, LRP1B, DLGAP1, MRPS27*
Cluster 7	*DAB2, AGFG1, SYT1, STON2, APOB, EPS15, LRP2*
Cluster 8	*ADCY3, PDE1A, PDE4D, PDE10A, ENTPD3, PDE4A, PDE4C*
Cluster 9	*RHOBTB3, SLC16A13, DENND4C, RIC1, DENND11, AVL9*
Cluster 10	*WARS2, AIMP2, AARS2, HARS2, FAM193B, EPRS1*
Cluster 11	*DMTN, EPB42, TRIM58, OSBP2, ATP8B4, FAM210B*
Cluster 12	*RALGAPA1, PLD1, VANGL1, CELSR3, SLK, CIT*
Cluster 13	*ATPAF1, FHOD3, GPR68, FHOD1, PTAFR*
Cluster 14	*METTL16, VWA7, IGF2BP3, YTHDF3, YTHDF1, FTO*
Cluster 15	*ANKRD24, TLE3, APC, BCL9L, TLE4, RFWD3*
Cluster 16	*C5AR1, C5, OPRM1, C3AR1, SSTR3, GPR37L1*
Cluster 17	*NVL, DIS3L, EXOSC2, PDCD11, PMEL*
Cluster 18	*HNF4A, MED23, MED18, PLPPR1, MED1*
Cluster 19	*CAPRIN1, PCBP2, EIF2A, G3BP1, FAM98A*
Cluster 20	*HSPA9, C20H3orf18, HGFAC, DNAJC22, EFHB*
Cluster 21	*SMG7, ZBTB37, FER, PLXNA4, AACS*

Table S3. Gene Ontology (GO) Cellular Component enrichment analysis for terms with FDR *p*-value <0.05.

Number of genes	Term name	Description	FDR value
56	GO:0005886	Plasma membrane	0.0236
13	GO:0036477	Somatodendritic compartment	0.0217
6	GO:0010494	Cytoplasmic stress granule	0.0095
32	GO:0042995	Cell projection	0.0052

FDR: False Discovery Rate

Table S4. Gene Ontology (GO) Molecular Function enrichment analysis for terms with FDR *p*-value <0.05.

Number of genes	Term name	Description	FDR value
3	GO:0008569	Minus-end-directed microtubule motor activity	0.0481
10	GO:0140098	Catalytic activity, acting on RNA	0.0328
8	GO:0016874	Ligase activity	0.0188
9	GO:0042277	Peptide binding	0.0182
17	GO:0140657	ATP-dependent activity	0.0038
29	GO:0032559	Adenyl ribonucleotide binding	0.0038
7	GO:0008081	Phosphoric diester hydrolase activity	0.0038

FDR: False Discovery Rate

Table S5. KEGG enrichment analysis for terms with FDR *p*-value <0.05.

Number of genes	Term name	Description	FDR value
4	cfa01522	Endocrine resistance	0.0457
8	cfa05014	Amyotrophic lateral sclerosis	0.045
6	cfa04020	Calcium signaling pathway	0.0405
4	cfa04911	Insulin secretion	0.0356
4	cfa05150	Staphylococcus aureus infection	0.0247
9	cfa04080	Neuroactive ligand-receptor interaction	0.0112
6	cfa04725	Cholinergic synapse	0.0056
5	cfa00071	Fatty acid degradation	0.0015
10	cfa04024	cAMP signaling pathway	2.00E-04
10	cfa05032	Morphine addiction	2.68E-07

FDR: False Discovery Rate

Table S6. Reactome pathway enrichment analysis for terms with FDR *p*-value <0.05.

Number of genes	Term name	Description	FDR value
3	CFA-202040	G-protein activation	0.042
8	CFA-373076	Class A/1 (Rhodopsin-like receptors)	0.0238
3	CFA-180024	DARPP-32 events	0.0171
5	CFA-373080	Class B/2 (Secretin family receptors)	0.0088
7	CFA-8856825	Cargo recognition for clathrin-mediated endocytosis	0.0015
6	CFA-191859	snRNP Assembly	1.60E-04
21	CFA-372790	Signaling by GPCR	5.65E-07

FDR: False Discovery Rate
